# Natural Extracts That Stimulate Adipocyte Browning and Their Underlying Mechanisms

**DOI:** 10.3390/antiox10020308

**Published:** 2021-02-17

**Authors:** Min-Kyeong Lee, Bonggi Lee, Choon Young Kim

**Affiliations:** 1 Department of Food Science and Nutrition, Pukyong National University, Nam-gu, Daeyeon Dong, Busan 608737, Korea; 3633234@hanmail.net; 2 Department of Food and Nutrition, Yeungnam University, Gyeongsan, Gyeongbuk 38541, Korea

**Keywords:** adipocyte browning, thermogenesis, edible dietary extracts, plant extracts, marine product extracts

## Abstract

Despite progress in understanding the developmental lineage and transcriptional factors regulating brown and beige adipocytes, the role of environmental modifiers, such as food components and natural extracts, remains to be elucidated. Furthermore, the undesirable pleiotropic effects produced by synthetic drugs targeting adipose tissue browning and thermogenesis necessitate research into alternative natural sources to combat obesity and related metabolic disorders. The current review, therefore, focused on the effects of various extracts from foods, plants, and marine products on adipose tissue browning and obesity. In particular, the recent findings of food components and marine products on adipose tissue browning will be discussed here.

## 1. Introduction

The World Health Organization estimated that 13% of the total adult population is currently obese, making it one of the most serious global health problems [1]. In addition, obesity is considered a primary factor contributing to the current epidemic of metabolic syndromes, including type 2 diabetes, hypertension, coronary artery diseases, brain disorders, and certain types of cancers [1]. Obesity results from adipocyte hypertrophy and hyperplasia caused by a severe energy imbalance between food intake and energy expenditure. Therefore, strategies aimed at reducing excess fat accumulation, especially in visceral regions, could ameliorate the current epidemic of metabolic disorders. Presently, the role of brown adipocytes in thermogenesis is a potential target for preventing obesity.

**Figure 1:**
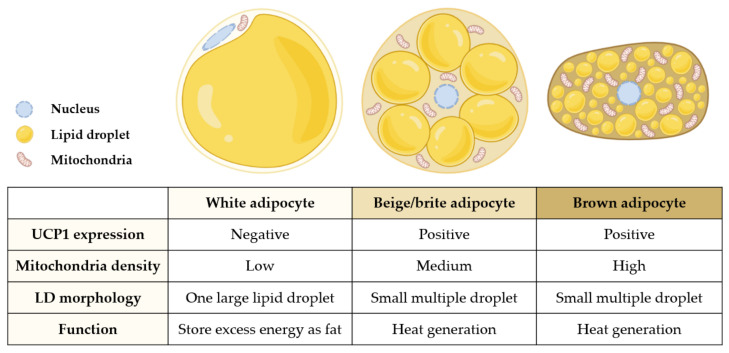
Characteristics of adipocytes. The figure represents distinct characteristics of white, beige, and brown adipocytes. UCP1, uncoupling protein 1; LD, lipid droplet.

Owing to the discovery of beige subtypes, adipocytes can now be categorized into white, beige, or brown adipocytes (Figure 1). White adipocytes have the function of storing excess energy as triglycerides and low mitochondrial density, whereas brown adipocytes dissipate energy as heat by combustion of nutrients in ATP generation via uncoupling protein 1 (UCP1) [2]. On the contrary, beige adipocytes exhibit both white and brown adipocyte characteristics. Beige adipocytes show a low basal expression of thermogenesis-related genes that are highly upregulated after thermogenic stimulations, including cold exposure and hormonal stimulations [3,4]. Although it has been a decade since the discovery of beige adipocytes, the origin of these adipocytes is still ill-defined. It is assumed that beige adipocytes can transdifferentiate from mature white adipocytes, white or brown adipocyte precursor cells, or specific beige adipocyte precursor cells [2].

**Figure 2:**
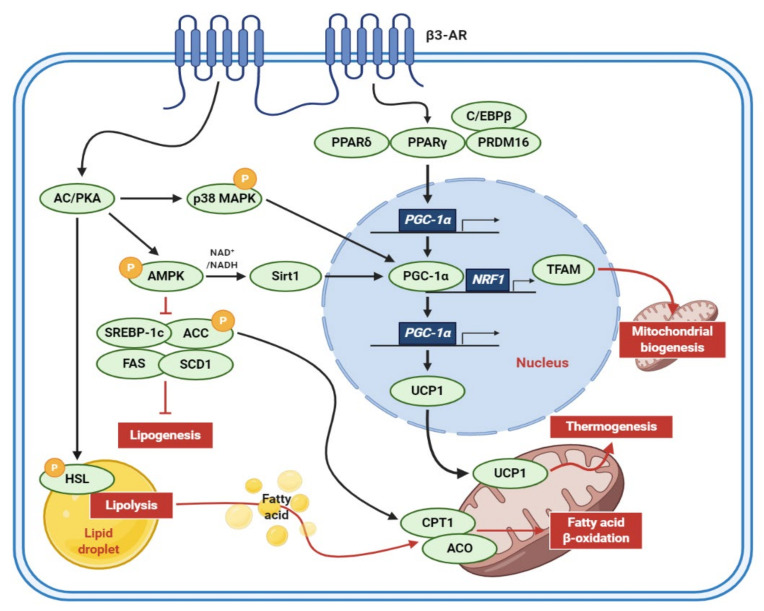
Molecular mechanisms of adipocyte browning. When stimulated by the catecholamines released from terminal neurons, β3-AR triggers the activation of signal transductions related to lipid catabolism and the browning of adipocytes. β3-AR, β3-adrenergic receptors; AC, adenylyl cyclase; PKA, protein kinase A; MAPK, mitogen-activated protein kinase; AMPK, AMP-activated protein kinase; NADH, nicotinamide adenine dinucleotide; Sirt1, sirtuin 1; SREBP-1c, sterol regulatory element-binding protein 1c; ACC, acetyl-CoA carboxylase; FAS, fatty acid synthase; SCD1, stearoyl-CoA desaturase 1; HSL, hormone-sensitive lipase; C/EBP, CCAAT/enhancer-binding protein; PPAR, peroxisome proliferator-activated receptor; PRDM16, PR/SET domain 16; PGC-1α, peroxisome proliferator-activated receptor-gamma coactivator-1-alpha; NRF1, nuclear respiratory factor 1-encoding gene; TFAM, mitochondrial transcription factor A; UCP1, uncoupling protein 1; CPT1, carnitine palmitoyltransferase 1; ACO, acyl-CoA oxidase.

It has long been suggested that human adults have brown adipocytes to elevate energy expenditure. Notably, the adult human brown adipocytes, which are primarily composed of beige adipocytes [3] are considered as a potential pharmaceutical target to fight obesity and related metabolic disorders [5]. The molecular mechanisms of adipocyte browning were elucidated in many in vivo and in vitro models (Figure 2). The murine 3T3-L1 preadipocytes are the cell model optimized for antiobesity studies due to well-established cellular and molecular mechanisms of adipocyte differentiation. When catecholamines, such as epinephrine and norepinephrine, are released from terminal neurons, the β3-adrenergic receptor (β3-AR) acts on adipocytes, activating the signal transductions involved in inducing a brown-like phenotype and enhancing lipid catabolism [6,7]. First, binding to β3-AR upregulates intracellular cyclic adenosine monophosphate (cAMP) levels and protein kinase A (PKA) activity, promoting lipolysis through the activation of hormone-sensitive lipase (HSL), which is a rate-limiting enzyme for lipolysis [6,8]. Activated PKA also induces the phosphorylation of AMP-activated protein kinase (AMPK), consequently suppressing sterol regulatory element-binding protein 1c (SREBP-1c), fatty acid synthase (FAS), stearoyl-CoA desaturase 1 (SCD1), acetyl-CoA carboxylase (ACC) but activating carnitine palmitoyltransferase 1 (CPT1), and acyl-CoA oxidase (ACO) to reduce lipogenesis and enhance fatty acid β-oxidation [6,7]. In addition, peroxisome proliferator-activated receptor gamma (PPARγ), peroxisome proliferator-activated receptor gamma coactivator-1-alpha (PGC-1α), and UCP1 are known to be sequentially activated by β-adrenergic signals to induce the browning of white adipocytes [6,7].

Various clinical studies have attempted to target beige adipocyte differentiation and function to ameliorate obesity, although the occurrence of undesirable side effects has led to the discontinuation of many molecules. For instance, catecholamines can activate beige adipocytes through β3-adrenergic receptor signaling pathways. However, several adrenergic ligands induce unfavorable autonomic, bone, and cardiovascular effects over time [5,9]. Similarly, other factors, including certain bone morphogenetic proteins, fibroblast growth factor 21 (FGF21), VEGFα, and atrial/brain-type natriuretic peptides have demonstrated their ability to stimulate beige adipocyte activation [5,9,10]. However, targeting these factors may also produce undesirable pleiotropic effects when developed into drugs [5]. For instance, FGF21 mimetics were withdrawn after phase 1 clinical trials because of their side effects [9]. Nevertheless, several clinical trials have shown beneficial effects of adipocyte browning to elevate energy expenditure and decrease fat mass [5,11,12].

To achieve both safety and multifunctionality, many in vitro and in vivo studies have focused on finding food extracts or natural products for stimulating adipocyte browning (elevating beige adipocyte differentiation and functions) and combating obesity and related metabolic diseases [13,14,15,16,17]. Therefore, this review will offer a critical evaluation of current findings on the potential roles of food-, plant-, and seaweed-derived extracts in adipocyte browning and obesity. The review will also cover active compounds within natural extracts that stimulate adipocyte browning and their underlying mechanisms.

## 2. Edible Dietary Extracts That Stimulate Adipocyte Browning

Antiobesity properties and multiple target mechanisms of edible dietary extracts have been reported. Recently, adipocyte browning is considered as one of the pivotal underlying mechanisms of antiobesity activities of extracts from fruits, tea, and legumes (Table 1).

### 2.1. Berries

Red and black raspberry (*Rubus coreanus* Miquel) is rich in health-beneficial phytochemicals with antioxidant activities [18]. Among the various health benefits, the antiobesity activity of raspberry has been reported.

In high-fat diet (HFD)-induced obese mice supplemented with red raspberry extract for 10 weeks, the extract inhibited body weight gain and white adipose tissue (WAT) accumulation but activated thermogenic gene (*Ucp1*, *Pgc1α*, and *CIDEA*) expression in brown adipose tissue (BAT) [19]. In addition, adipogenic and mitochondria-related genes were upregulated in BAT of mice administrated with red raspberry extracts. Moreover, this extract induced brown adipogenesis and thermogenic gene expression in primary brown preadipocytes.

The activation of adipocyte browning by black raspberry has been reported in both in vitro and in vivo models [17]. While black raspberry extract suppressed white adipocyte differentiation in human mesenchymal cells, murine 3T3-L1 preadipocyte, and zebrafish, adipocyte browning was activated by upregulating beige adipocyte-specific markers, including UCP1, PGC1α, NRF1, CIDEA, and CPT1B. Similarly, the gene expression of beige-specific markers such as UCP1, PRDM16, PGC1α, and TBX1 was induced in inguinal WAT in cold-stressed mice fed with black raspberry extract for 2 weeks. Based on HPLC-MS/MS analysis, 4-hydroxybenzoic acid, ellagic acid, gallic acid, and salicylic acid were identified in black raspberry extract. Among them, ellagic acids in black raspberry responsible for adipocyte browning as demonstrated by the 3T3-L1 cell experiment.

Strawberry (*Fragaria* × *ananassa*) exhibits health-promoting and preventive activities against various diseases, including cancer, cardiovascular diseases, type 2 diabetes, and obesity [27]. Inhibition of lipid accumulation and induction of adipose browning by strawberry extract was demonstrated in 3T3-L1 cells [20]. Strawberry extract reduced intracellular lipid accumulation by downregulating adipogenic transcription factors such as *PPARγ* and *C/EBPα*. Meanwhile, BAT-specific genes (*Pdk4* and *Ucp1*) were stimulated, suggesting adipocyte browning. Furthermore, strawberry extract elevated the levels of proteins related with mitochondrial biogenesis such as AMPKα, sirtuin 1, and PGC-1α [20]. Consistent with these gene and protein expression profiles, oxygen consumption rate and uncoupled respiration were also increased by strawberry. 

### 2.2. Omija Fruit

Omija (*Schisandra chinesis*), a magnolia berry with five different distinct flavors (sourness, sweetness, bitterness, pungentness, and saltiness) showed antioxidant and anti-inflammatory activities [28]. The inhibitory effects of omija extract on obesity have been shown in 3T3-L1 cells and rats [21]. Since the adipogenic transcription factors of *C/EBPβ, PPARγ*, and *C/EBPα* were significantly decreased, omija extract treatment suppressed adipogenesis, and HFD-induced adipose tissue accumulation and body weight gain. Park et al. [22] showed that omija supplementation (500 mg/kg body weight) for 16 weeks reduced epididymal and visceral WAT but increased BAT in HFD-induced obese mice. Indirect calorimetry analysis also showed significant energy expenditure in omija-treated mice [22]. Consistent with these results, epididymal WAT exhibited beiging indicated by the expression of BAT-specific genes, such as *Pparα*, *CIDEA*, and cytochrome c oxidase subunit 8 B (*COX8β*). In addition, omija feeding improved hepatic steatosis, dyslipidemia, and glucose tolerance. These results suggest that omija extract helps to prevent obesity and metabolic disturbances by activating the browning of white adipocytes.

### 2.3. Green Tea

Green tea (*Camellia sinensis*), the second most popular consumed drinks in the world, showed potent antiobesity effects through the suppression of food intake, dietary fat bioavailability, adipogenesis, fat synthesis, together with accelerated fat oxidation, fecal lipid excretion, and energy expenditure by thermogenesis [29]. Neyrinck et al. [16] demonstrated the antiobesity activity of green tea extract by activating adipocyte browning. HFD-fed mice supplemented with 0.5% green tea leaf extract for 8 weeks had significantly lower body weight and white and brown adipocytes weights [16]. In addition, the levels of genes related to mitochondrial activity (*Pgc1α* and *Cpt1b*) and brown-specific markers (*Ucp1*, *Prdm16*, and *CIDEA*) were higher in the subcutaneous WAT of mice fed with green tea and HFD compared with mice fed with HFD [16]. Additionally, immunohistological analysis of UCP1 in the subcutaneous WAT of the green tea-supplemented mice showed a significantly higher staining area than that of untreated mice, confirming the browning of WAT.

### 2.4. Cinnamon

Cinnamon is a spice derived from the inner bark of trees from the genus *Cinnamomum cassia* belongs to the Lauraceae family. Cinnamon has been used as a culinary ingredient and traditional medicine due to its antioxidant, anti-inflammatory, antihyperglycemic, and antihyperlipidemic activities [30]. Recently, the stimulatory effect of cinnamon extract on the activation of BAT and the browning of WAT. Kwan et al. showed that the treatment of cinnamon extract elevated the gene expression of brown adipocyte markers (*Cidea, Prdm16*, *Pgc,* and *Cpt-1)* while reduced that of white adipocyte markers (*Dpt* and *Igf*) in 3T3-L1 adipocytes and WAT from *db/db* mice. In the same study, HFD-induced obese mice were fed with cinnamon extract exerted significantly lower body weight and elevated brown adipocyte marker genes such as *Ucp1*, *Cidea*, and *Prdm16* in WAT. Another study showed that cinnamon extract stimulates thermogenesis in mice under cold exposure by the activation of brown adipose tissue [24]. While mice placed in cold environments reduced body temperature and energy expenditure, mice fed with cinnamon extract increased those by activation of BAT (upregulation of UCP1 and PGC-1α) and browning of WAT (upregulation of *Ucp1*, *Ppargc1α*, and *Prdm16*). The underlying mechanisms of cinnamon extract were determined as the activation of the AMPK/SIRT1 pathway [24].

### 2.5. Germinated Soybean Germ

Germination enhances phytochemicals’ health-promoting activities such as antioxidant and anti-inflammatory properties [31]. Kim et al. [25] reported that germinated soy germ extract prevents obesity through adipocyte browning in vitro and in vivo. In 3T3-L1 cells, germinated soybean germ treatment lowered accumulation of lipids and expression levels of genes involved in adipogenesis (C/EBPβ, PPARγ, and C/EBPα) and lipogenesis (SREPB1c, ACC, FAS, and SCD1) but elevated those of genes involved in lipolysis, β-oxidation, and thermogenesis. Consistent with the in vitro results, germinated soybean germ extract reduced body weight gain and adipose tissue accumulation in mice by inhibiting genes related to adipogenesis and lipogenesis. Germinated soybean germ extract also induced adipocyte browning by upregulating UCP1, CPT1, PGC-1α, and PPARα.

### 2.6. Ganoderma Tsugae

*Ganoderma tsugae* Murrill is an edible fungi in the family Ganodermataceae. It is also known as lingzhi or reishi and is a traditional Chinese medicinal mushroom used as a popular functional food worldwide [32]. Various pharmacologically active ingredients, such as polysaccharides, triterpenoids, nucleosides, proteins/peptides, organic germanium compounds, and other trace elements, have been isolated from *G. tsugae* extracts and characterized [32]. These active ingredients have demonstrated several therapeutic effects, including antioxidant, anti-inflammatory, antibacterial, antitumor, anti-HIV, blood pressure regulatory, hepatoprotective, and immunomodulatory, cardioprotective properties and can act as a nerve tonic [33,34,35].

In addition to these therapeutic effects, Tseng et al. [26] recently demonstrated that triterpenoid-rich *G. tsugae* ethanol extract (GTEE) could improve obesity by promoting intracellular metabolic flexibility and plasticity and inducing adipocyte browning. The data showed that low-dose GTEE (0.2 mg/mL) in 3T3-L1 cells induced adipogenesis and small lipid droplet formation and promoted intracellular lipid metabolism flux and flexibility [26]. It also induced the browning of 3T3-L1 adipocytes by upregulating UCP1, CIDEA, HSP60, and cytochrome c proteins while downregulating the NADH/NAD^+^ ratio and NADH content [26]. Furthermore, in vivo investigations of GTEE administration to male C57BL/6Narl HFD-fed mice upregulated UCP1 in the inguinal WAT to induce browning while reducing fasting blood glucose, triglyceride, and LDL cholesterol levels, thereby improving glucose intolerance and lipid dysfunction [26]. These effects of GTEE were accompanied with increased expression level of SIRT1 and threonine-172 phosphorylation of AMPK. This indicates GTEE as a prospective treatment strategy for obesity.

## 3. Plant Extracts That Stimulate Adipocyte Browning

Emerging research indicates that plant extracts can be important environmental regulators of WAT browning and energy homeostasis. Therefore, various in vitro and in vivo studies have investigated the effects of plant extracts on adipocyte metabolism and obesity (Table 2).

### 3.1. Panax Ginseng

Ginseng (*Panax ginseng* Mayer) of the Araliaceae family is a traditional medicinal herb registered in the Chinese classic oriental herbal dictionary as “shin-nong-bon-cho-kyung” with various pharmacological properties [36]. Depending on the processing method, ginseng can be classified into three types: white, red, and black [36,37]. Among these, black ginseng (BG), a ginseng product prepared by the steaming-and-drying process for nine times, exhibits more potent pharmacological and biological activity than either white or red ginseng [36,37,38]. This difference may be because of the conversion of ginsenosides from fresh ginseng into less polar forms during steaming [38]. To date, 19 types of ginsenosides (F4, Rb1, Rb2, Rc, Rd, Re, Rf, Rg1, Rg5, Rg6, Rh4, Rk1, Rk3, Rs4, Rs5, 20(R)-Rg3, 20(R)-Rs3, 20(S)-Rg3, and 20(S)-Rs3) have been identified in BG [37,38]. Previous studies have demonstrated that ginsenosides Rb1, Rb2, Rg1, and Rg3 exhibit antiobesity activity [39,40,41,42,43].

Park et al. [43] showed that BG and ginsenoside Rb1 exhibit antiobesity effects by inducing browning in 3T3-L1 adipocytes and primary white adipocytes (PWATs) through activation of the AMPK signaling cascade. BG (25, 50, and 100 μg/mL) and Rb1 (10, 20, and 40 μM) inhibited the early stages of adipogenesis in 3T3-L1 adipocytes and PWATs by lowering the expression levels of C/EBPα and SREBP-1c to reduce lipid accumulation [43]. In addition, BG and Rb1 have been shown to induce browning of 3T3-L1 adipocytes and PWATs through increased mitochondrial activity and expression of BAT-specific markers such as PRDM16, PGC-1α, PPARγ, and UCP1 [43]. However, this phenomenon was abolished in PWATs treated with the AMPK inhibitor dorsomorphin, suggesting that the browning and antiobesity effects of BG and Rb1 are mediated by activation of the AMPK signaling cascade [43].

Another study investigated the antiobesity activity of ginsenoside Rb2 [39]. Data showed that Rb2 (40 mg/kg/day) treatment resulted in lower body weight gain, enhanced insulin sensitivity, and elevated energy expenditure in diet-induced obese (DIO) mice [39]. Additionally, treating mice with Rb2 was shown to induce the activation of BAT and browning of WAT by reducing lipid droplet size, stimulating UCP1 staining, and upregulating thermogenic and mitochondrial gene expression [39]. The authors also reported that activation of the AMPK pathway is responsible for Rb2-mediated upregulation of browning gene expression [39]. Furthermore, in other studies, Lee et al. and Kim et al. [40,41] demonstrated that Rg1 and Rg3 exert anti-obesity effects by upregulating browning-related gene expression and mitochondrial biosynthesis through activation of the AMPK pathway.

These results suggest the potential for BG and ginsenosides to act as browning agents and exert anti-obesity effects, an area that may be explored in future studies. Further investigation is still needed to confirm whether Rb1, Rb2, Rg1, and Rg3, and other ginsenosides, contribute to the browning process.

### 3.2. Phyllostachys Pubescens and Scutellaria baicalensis

The leaves of *Phyllostachys pubescens* Mazel (bamboo), a plant of the family Poaceae commonly consumed as a tea, have various biological functions, such as being an antioxidant and anticoagulant [47]. The roots of *Scutellaria baicalensis* Georgi (Baikal skullcap), a plant of the family Lamiaceae, are widely used in traditional medicine, preventing inflammation, diuresis, and diarrhea [48].

In a recent study, a 2:1 (*w:w*) mixture of *P. pubescens* leaf extract and *S. baicalensis* root extract (BS21) was used to investigate its effects on adipogenesis and browning in vitro [49]. The results showed that BS21 treatment markedly reduced the accumulation and size of lipid droplets and the levels of leptin and adiponectin in 3T3-L1 adipocytes [49]. In addition, treatment with BS21 lowered the expression levels of white adipocyte markers (PPARγ, C/EBPα, and ap2) and lipogenic markers (SREBP1c and FAS), but increased the expression levels of β-oxidation proteins (CPT1 and p-ACC) [49]. Furthermore, BS21 treatment increased the expression of brown adipocyte marker proteins, such as UCP1, PRDM16, and PGC1α, and thermogenesis-related genes such as *UCP2* in 3T3-L1 adipocytes, suggesting that BS21 induces adipocyte browning and thermogenesis [49]. These effects were mediated by activation of the AMPK signaling pathway, indicated by Western blot analysis [49]. According to UPLC analysis, chlorogenic acid, orientin, baicalin, wogonoside, baicalein, tricin, wogonin, and chrysin were identified in BS21, of which adipocyte differentiation and leptin level were markedly suppressed in all compounds except for wogonoside [49]. These results suggest that BS21 has antiobesity efficacy by inhibiting adipogenesis and lipogenesis and inducing fat oxidation and browning.

### 3.3. Humulus Japonicus

*Humulus japonicus*, also known as “Japanese hop”, is a perennial herb belonging to the Cannabaceae family and is ubiquitously found in Asian countries, including Korea, Japan, and China [50]. *H. japonicus* has long been used in traditional medicine to cure skin diseases, pulmonary tuberculosis, and hypertension [50]. Other biological effects of *H. japonicus* include antimutagenic, antibacterial, antiatheroma, antioxidant, antitumor, antimycobacterial, and anti-inflammatory effects [50,51,52,53,54].

A recent study has shown that *H. japonicus* aqueous extract (AH) can suppress obesity by stimulating thermogenesis and improving oxidative stress through a BAT-like phenotype in 3T3-L1 adipocytes [44]. Treatment with AH (20 and 100 μg/mL) upregulated the expression of BAT-specific markers, such as UCP1, PRDM16, and PGC-1α, in 3T3-L1 adipocytes, suggesting that AH stimulates thermogenesis and browning [44]. In addition, AH promoted fatty acid oxidation and lipolysis while notably inhibiting lipogenesis and lipid accumulation [44]. Oxidative stress could influence the development of obesity-related metabolic disorders [55]. AH also improved hydrogen peroxide-induced oxidative stress and markedly increased the expression level of the antioxidant enzymes SOD1, catalase, and GPx1 [44]. The antiobesity process of stimulating browning and lipid metabolism while reducing oxidative stress in WAT has been shown to involve activation of the AMPK and PPARδ signaling pathways [44]. However, more research into the stimulation of thermogenesis and induction of oxidative stress improvement by *H. japonicus* is needed using in vivo models of obesity.

### 3.4. Immature Citrus Reticulata

Ponkan (*Citrus reticulata*) is a commercial Mandarin cultivar belonging to the genus *Citrus* of the Rutaceae family [56]. Immature *C. reticulata* contains higher amounts of p-synephrine, nobiletin, and tangeretin than other citrus species, showing diverse biological activities [57].

In rodents, immature *C. reticulata* extract (ICRE) can exert positive effects on browning of WAT, thereby suppressing obesity and hepatic steatosis [45]. Administration of 1% ICRE for 11 weeks induced body weight loss in HFD-fed mice, accompanied by a decrease in visceral fat (epididymal, retroperitoneal, and mesenteric) weight and adipocyte size [45]. The administration of ICRE also markedly reduced hepatic steatosis, fasting blood glucose, insulin, and homeostatic model assessment for insulin resistance, serum triglyceride, and total cholesterol levels, suggesting that ICRE can improve HFD-induced fatty liver disease, insulin resistance, and dyslipidemia [45]. In particular, the administration of ICRE improved cold tolerance during cold exposure in HFD mice. These effects were associated with the increased expression level of thermogenic genes, such as *UCP1*, *PRDM16*, and *NRF1*, and beige adipocyte-selective markers, such as TEME26, CD137, and CIDEA, in inguinal WAT. These results suggest that ICRE has antiobesity efficacy by inducing BAT-like formation. However, more clarity is need on the possible molecular mechanisms involved in ICRE-induced adipocyte browning.

### 3.5. Glucoraphanin from Broccoli Seeds

Glucoraphanin, a precursor of sulforaphane, is an isothiocyanate derivative found in cruciferous vegetables of the family Brassicaceae, such as cauliflower, collard, and cabbage, and especially highly concentrated in broccoli seeds and sprouts [58]. When plant tissues and cells are destroyed by external stimuli, glucoraphanin is hydrolyzed by myrosinase to produce biologically activated sulforaphane [59]. Sulforaphane is a potent natural nuclear factor (erythroid-derived 2)-like 2 (Nrf2) inducer that has various pharmacological effects, such as antioxidant, anticancer, anti-aging, and anti-inflammatory effects [60].

It has recently been confirmed that glucoraphanin can induce browning of adipose tissue and reduce metabolic endotoxemia, thereby preventing metabolic disorders, such as obesity and type 2 diabetes [46]. In HFD-induced obese mice, administration of 0.3% glucoraphanin for 14 weeks reduced body weight gain and fat mass and increased energy expenditure. Glucoraphanin also increased the expression level of UCP1, a major browning marker, in inguinal and epididymal adipose depots and improved systemic glucose tolerance and insulin sensitivity. However, this phenomenon was abolished in homozygous NRF2-deficient (Nrf2^–/–^) mice, suggesting that the antiobesity and insulin-sensitizing effects of glucoraphanin are mediated by NRF2. Additionally, glucoraphanin downregulated plasma lipopolysaccharide levels and reduced the relative abundance of Desulfovibrionaceae bacteria, which are major producers of endotoxins in the gut microbiome. It also inhibited hepatic lipogenic gene expression, lipid peroxidation, M1-like macrophage accumulation, and inflammatory signaling pathways, suggesting that glucoraphanin relieves oxidative stress and inflammation caused by HFD. 

Taken together, these findings indicate that glucoraphanin may be effective in preventing hepatic steatosis, insulin resistance, and chronic inflammation by promoting energy expenditure and suppressing gut-derived metabolic endotoxemia.

## 4. Marine Products That Induce Adipose Browning

Marine products have great attention due to their potential applications in functional foods and food additives because of their health-promoting properties, including antiobesity, antioxidant, anti-inflammatory, and anticancer effects [61]. Recent studies have revealed new roles of marine products in adipocyte browning and energy balance (Table 3).

### 4.1. Sargassum Serratifolium (C. Agardh)

*Sargassum serratifolium*, a marine brown alga, has been widely utilized as a food and traditional medicine in Asia. A study examined the roles of a meroterpenoid-rich fraction of an ethanolic extract of *S. serratifolium* (MES) in HFD-induced obesity and related metabolic syndrome C57BL/6J mice. MES administration notably decreased obesity and hepatic steatosis without altering food intake. The beneficial effects of MES are partially ascribed to increased lipid catabolic AMPK signaling pathways and highly elevated UCP1-positive cells in adipose tissue [14], indicative of MES-stimulated adipose tissue browning.

Chemical composition analysis revealed that MES contains a high level of meroterpenoids, including sargachromenol, sargaquinoic acid (SQA), and sargahydroquinoic acid (SHQA) as the most abundant compound [14]. When the direct effects of SHQA on adipocytes were tested in conditions simulating adipocyte browning, lipolysis, and mitochondrial number was increased, but ATP production decreased. The authors further examined uncoupled metabolic signaling and found that SHQA treatment significantly elevated UCP1-positive adipocytes and beige adipocyte markers [62]. The adipose browning effects of SHQA were related to the activation of PPARγ, PPARα, and AMPKα.

The browning effects of SQA were tested using 3T3-L1 adipocytes differentiated under probrowning conditions. SQA treatment stimulated the differentiation of white adipocytes into beige adipocytes and decreased lipid accumulation. SQA activated AMPK and upregulated genes associated with lipid catabolic pathways, including perilipin, carnitine palmitoyltransferase 1, and acyl-CoA synthetase long-chain family member 1 [13]. These findings confirm SHQA and SQA as active MES compounds that stimulate lipid catabolic signaling and facilitate white to beige adipocyte differentiation. Further studies are still required on the adipocyte browning effects of sargachromenol, the remaining major MES compound.

**Table 3:** Marine products to stimulate adipocyte browning.

Extract (Part/Solvent)	Model	Conc.	Effects	Active Component	Ref.
*Sargassum* *Serratifolium* (whole/EtOH*)*	C57BL/6J mice	30–120 mg/kg/day	↑AMPK signaling pathway ↑UCP1-positive cells ↓lipogenesis (↓SREBP1c, SCD-1, FAS) ↑lipolysis (↑PLIN, CPT1, ACSL1) ↑mitochondria function	SHQA SQA	[13,62]
*Spirulina maxima* (whole/EtOH)	3T3-L1 cells	50, 100 μg/mL	↑lipid accumulation ↓adipogenesis (↓C/EBPα, PPARγ, aP2) ↓lipogenesis (↓SREBP1c, ACC, FAS, LPAATβ, Lipin1, DGAT1)	chlorophyll A C-phycocyanin	[63]
	ICR mice	150, 450 mg/kg/day	↑p-AMPK ↑adipose browning proteins (↑PRDM16, PGC1α, UCP1)		
*Phaeodactylum* *Tricornutum* (whole/ND)	C57BL/6J mice	0.81, 1.62, 3.25 mg/kg/day	↓body weight, organ weight, adipocyte size ↑blood metabolic profile	fucoxanthin	[64]
	3T3-L1 cells	20, 40 μM	↑UCP1		
*Nitzschia laevis* (whole/EtOH)	C57BL/6J mice	10, 50 mg/kg/day	↓body weight↑BAT cell number ↑thermogenesis (↑UCP1)	ND	[65]
*Undaria pinnatifida* (whole/chloroform/ methanol)	Wistar rats and KK-Ay mice	0.5 and 2% in diet	↓WAT weight ↑BAT weight ↑UCP1 only in WAT	fucoxanthin	[66]

AMPK, AMP-activated protein kinase; UCP1, uncoupling protein 1; SREBP1c, sterol regulatory element-binding protein 1c; SCD1, stearoyl-CoA desaturase 1; FAS, fatty acid synthase; PLIN, perilipin; CPT1, carnitine palmitoyltransferase 1; ACSL1, acyl-CoA synthetase long-chain family member 1; SHQA, sargahydroquinoic acid; SQA, sargaquinoic acid; C/EBPα, CCAT/enhancer binding protein alpha; PPARγ, peroxisome proliferator-activator receptor gamma; aP2, adipocyte protein 2; ACC, acetyl-CoA carboxylase; LPAATβ, lysophosphatidic acid acyltransferase beta; DGAT1, diacylglycerol acyltransferase 1; PRDM16, PR domain zinc finger protein 16; PGC1α, peroxisome proliferator-activated receptor-gamma coactivator-1-alpha; BAT, brown adipose tissue. ND, not determined.

### 4.2. Spirulina Maxima

*Spirulina maxima* is a microalga that is abundant in essential nutrients and includes pigment proteins including chlorophyll a and C-phycocyanin [67,68]. Studies have reported that *S. maxima* exhibit antioxidant, anticancer, and neuroprotective effects [61] but its potential in obesity and adipose tissues is unclear. A recent study examined the ethanol extract of *S. maxima* on adipogenesis, lipogenesis, and browning using in vitro and in vivo mouse models [63]. The treatment of the extract decreased intracellular lipid accumulation in 3T3-L1 adipocytes, reduced protein expression levels of the adipogenic genes, including *C/EBPα, PPARγ,* and *aP2*, and the lipogenic genes, including *SREBP1, ACC, FAS, LPAATβ, Lipin1,* and *DGAT1* [63]. When the extract was supplemented in HFD-fed male ICR mice, DIO was inhibited at both low and high concentrations (150 and 450 mg/kg, respectively). Furthermore, *Spirulina* ethanol extract-supplemented mice showed lower adipose tissue mass and blood lipid concentrations than HFD-fed mice. These characteristics are closely related to the activation of AMPK and the upregulation of adipose browning proteins, including PRDM16, PGC1α, and UCP1 [63]. When the index components (chlorophyll A and C-phycocyanin) of the ethanol extract were administered in 3T3-L1 and C3H10T1/2 cells, both compounds decreased the protein expression related to adipogenesis and lipogenesis. However, their effects on browning markers still need to be confirmed in future studies.

### 4.3. Phaeodactylum Tricornutum

*Phaeodactylum tricornutum* is a plentiful source of fucoxanthin, a marine carotenoid found in macroalgae, with health-promoting effects, including antiobesity effects. However, fucoxanthin is scarce and requires delicate extraction techniques, limiting the number of applied research studies to date [64]. In one such study, the antiobesity effects of a *Phaeodactylum* ethanol extract were tested using cell and animal models. In 3T3-L1 adipocytes treated with the extract, UCP1 protein levels were upregulated at 400 μg/mL. When applied to HFD-induced obese mice, the oral injections of *Phaeodactylum* extract (0.81, 1.62, and 3.25 mg/kg/day) reduced body weight, organ weight, and adipocyte size while ameliorating the blood metabolic profile [64]. In the same study, fucoxanthin treatment at 20 or 40 μM also upregulated the protein levels of UCP1 in adipocytes. When orally injected into the HFD mice, fucoxanthin (0.1 mg/kg/day) ameliorated DIO and metabolic profile. These findings suggest that fucoxanthin mediates the adipose browning and antiobesity effects of *Phaeodactylum* extract.

### 4.4. Nitzschia Laevis

The microalga *Nitzschia laevis* has been reported to contain polyphenols, polyunsaturated fatty acids, and fucoxanthin [65,69,70]. *Nitzschia laevis* extract has great potential as dietary supplement for various health-promoting effects, and its effects on obesity have also recently been confirmed. An ethanol extract of *N. laevis* (10 or 50 mg/kg/day) was administered orally to HFD-fed C57BL/6J mice, significantly decreasing body weight without altering food intake and lipid accumulation in the liver and WAT [65]. In addition, supplementation with *N. laevis* significantly elevated the number of BAT cells and the mRNA expression levels of *UCP1*. In the same study, *N. laevis*-fed mice maintained gut epithelium integrity and gut microbiota composition against HFD-induced complications. Although the focus of the study was largely on the effects of *N. laevis* on the composition of the gut microbiota’s role in antiobesity, an UCP1-induced thermogenic effect by *N. laevis* supplementation is still plausible. Further research into the roles of *N. laevis* supplementation in the browning of WAT and related signaling pathways are still required.

### 4.5. *Undaria* Pinnatifida

*Undaria pinnatifida* is one of the most consumed edible seaweed in Korea and Japan. A study examined the antiobesity effects of lipids from *Undaria pinnatifida* using male Wistar rats and female KK-Ay mice. When the animals were fed 2% *Undaria* lipids mixed with experimental diets, the weight of WAT was significantly reduced while the weight of BAT was greater than in that of control mice [66]. Nevertheless, there was no difference in *UCP1* expression in BAT between groups, indicating that the reduction in WAT weight in *Undaria* lipids-fed mice may not be explained only through the UCP1-dependent thermogenic energy expenditure in BAT. Thus, the authors tested whether *Undaria* lipids affect thermogenic signaling in WAT of mice [66]. While there was little expression in the WAT of control mice, the protein levels of UCP1 markedly increased in WAT of *Undaria* lipids-fed mice in a concentration-dependent manner. However, UCP2 expression was reduced by *Undaria* lipid feeding compared to the control mice, suggesting that UCP1 signaling in WAT contributes to the significant decrease in fat mass in *Undaria* lipids-fed mice [66]. Fucoxanthin may be involved in the *Undaria* lipids-mediated antiobesity activity [64,66]. When dried powder of *Undaria* *pinnatifida* after removing carbohydrate and protein was extracted with chloroform/methanol (2:1, *v/v*), the powder contained 15% (*w/w*) lipids, of which fucoxanthin was quite abundant (9.6%) [66]. However, conflicting results also exist when fucoxanthin was treated in human adipocytes [71]. When human adipocytes were treated with fucoxanthin or fucoxanthinol because fucoxanthin is converted to fucoxanthinol in adipocytes of mice in 48–57 h [72], there were no apparent effects on oxygen consumption rate and the mRNA expression levels of genes related to adipocyte browning including *UCP1* and *PGC1α* [71]. Therefore, further studies are necessary to determine the active components of *Undaria* lipids that stimulate adipocyte browning and antiobesity effects.

## 5. Conclusions

**Figure 3:**
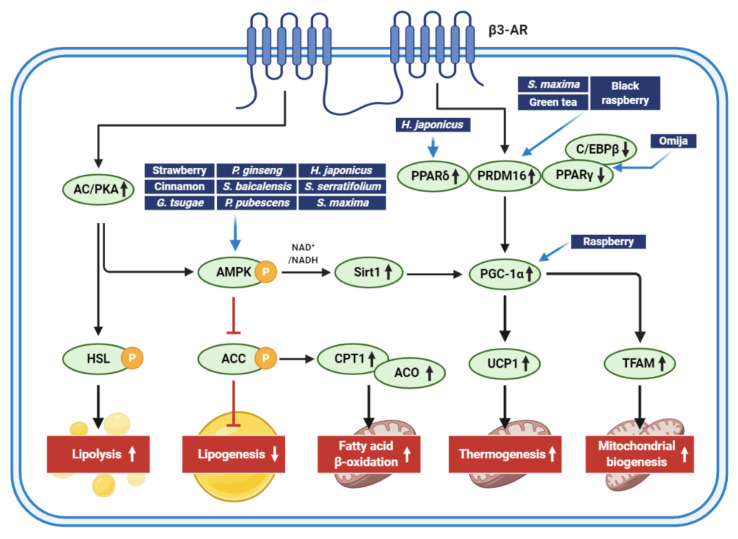
Signaling pathways regulated by extracts isolated from edible foods, plants, and marine products for adipocyte browning and antiobesity activities. The figure shows major pathways inducing lipid catabolism and mitochondrial functions that aid in weight loss. Although key molecules regulated by edible foods, plant extracts, and marine products for adipocyte browning and antiobesity effects are shown here, it should be noted that many extracts can, directly and indirectly, control multiple signaling pathways that regulate energy balance.

Natural extracts are of specific interest in the prevention and management of obesity. Accumulated evidence supports that natural extracts that stimulate adipocyte browning may help manage obesity through a variety of mechanisms. Figure 3 presents an overview of the cellular targets of natural extracts that induce adipocyte browning. The extracts from edible foods, plants, and marine products regulate lipid metabolisms, thermogenesis, and mitochondrial biogenesis.

Based on the studies covered by this review, many edible foods, plant extracts, and marine products have stimulated adipocyte browning and altered energy balance by regulating key molecules involved in adipogenesis, lipid catabolism, and mitochondrial functions. However, most of the data were derived from mouse-originated cells and animal models, with established higher temperature homeostasis than humans, contributing to a higher sensitivity to induce adipocyte browning. It should be noted that studies from cell models and animal models do not always correspond to each other possibly due to low bioaccessibility and bioavailability of many natural compounds. Therefore, it may be possible that natural extracts with little effects on adipocyte browning in animal studies may have better effects when directly treated in cell models. Additionally, we cannot assure that natural extracts directly regulate adipocyte metabolism based on animal studies because there are multiple tissues and hormones that are involved in adipocyte browning. Nevertheless, many natural extracts exhibited promising effects on thermogenesis and obesity. To further develop natural extracts as functional foods, clinical trials are necessary to confirm the efficacy and safety.

## Figures and Tables

**Table 1:** Stimulatory effects of edible extracts on adipocyte browning.

Extract (Part/Solvent)	Model	Conc.	Effects	Active Component	Ref.
Red raspberry (fruit/hot water)	C57BL/6J mice	100 mg/kg/day	↓obesity (↓body/ WAT weights) ↑thermogenesis (↑*ucp1, pgc1α, Cidea*)	ellagic acid	[19]
			↑mitochondrial biogenesis (↑*Sirt3, Nrf1*)		
	Primary brown preadipocytes	100 μg/mL	↑mitochondrial activity ↑brown adipogenesis (*ap2, adiponectin, resistin*) ↑thermogenesis (↑*ucp1, pgc1α, Cidea*) ↑p-AMPK, p-ACC		
Black raspberry (fruit/hot water)	hMSCs	5–10 μg/mL	↓adipogenesis	ellagic acid	[17]
	zebrafish	100 μg/mL	(↓PPARγ, C/EBPα)		
	3T3-L1 cells	5–10 μg/mL	↓adipogenesis (↓PPARγ, C/EBPα) ↑thermogenesis (↑UCP1, PGC1α) ↑mitochondrial function (↑CIDEA, Nrf1, CPT1B)		
	C57BL/6J mice	100 mg/kg/day	↑thermogenesis (↑body temperature in cold-exposure, ↑UCP1, PGC1α, PRDM16, TBX1)		
Strawberry (fruit/80% MeOH)	3T3-L1 cells	5–10 μg/mL	↓adipogenesis (↓*PPARγ, C/EBPα, resistin*) ↑thermogenesis (↑*UCP1, PDK4*) ↑mitochondrial biogenesis (↑AMPK, Sirt1, PGC1α)	ND	[20]
Omija fruit (fruit/50% EtOH)	3T3-L1 cells Sprague–Dawley rats	20, 150 μg/mL 5, 200 mg/kg/day	↓adipogenesis (*↓C/EBPβ, PPARγ, C/EBPα*) ↓obesity (↓body/ WAT weights)	ND	[21]
	C57BL/6J mice	500 mg/kg/day	↓WAT /↑BAT weights ↑energy expenditure ↑thermogenesis (↑*Pparα, Cidea, and COX8β.*)	ND	[22]
Green Tea (leaf/water)	C57BL/6J mice	0.5%	↓obesity (↓body/ WAT/ BAT weights) ↑brown-specific markers (↑*Ucp1, Prdm16*, and *CIDEA*) ↑mitochondrial activity (↑*Pgc1α* and *Cpt1B*)	Catechin EGCG	[16]
Cinnamon (bark/70–80% EtOH)	3T3-L1 cells	80 μg/mL	↑brown-specific markers (↑*Cidea, Prdm16*, *Pgc, Cpt-1* and ↑PRDM16), cAMP ↓white adipocyte markers (↓*Dpt* and *Igf*)	Catechin Quercetin Icariin Chlorogenic acid Protocatechuic acid asculetin	[23]
	ex vivo adipocytes isolated from *db/db* mice	80 μg/mL	↑brown-specific markers (↑*Ucp-1, Cidea, Prdm16* and ↑UCP1)		
	C57BL/6J mice fed with high fat diet	500 mg/kg/day	↑brown-specific markers (↑*Ucp-1, Cidea, Prdm16* and ↑UCP1) ↓white adipocyte markers (↓*Dpt*)		
	Kunming mice	90, 180, 360 mg/kg/day	↑thermogenesis (↑body temperature in cold-exposure, ↑*Ucp1, Ppargc1 α, Prdm16 in BAT*) ↑energy expenditure (↑VO_2_ and VCO_2_) Uncoupling ATP production, AMPK-SIRT1 pathway in BAT	Cinnamaldehyde Cinnamic acid 2-methoxycinnamaldehyde coumarin	[24]
Geminated soy germ(germ/EtOH)	3T3-L1 cells	0.1–10 μg/mL	↓adipogenesis, lipogenesis ↑Lipolysis, β-oxidation	Soya saponin Ab	[25]
	C57BL/6J mice	1 mg/kg	↓obesity (↓body/ WAT weights) ↑thermogenesis ↑mitochondrial biogenesis		
*Ganoderma tsugae* (fruiting body/EtOH)	3T3-L1 cells	0.2 mg/mL	↑small lipid droplets formation ↑intracellular lipid metabolism flux/flexibility ↑UCP1, Cidea, HSP60, cyto *c* ↓NADH/NAD^+^ ratio, NADH content	Triterpenoid	[26]
	C57BL/6Narl	150, 300 mg/kg/day	↑WAT browning (↑UCP1)↓glucose/lipid disorders ↑SIRT1, p-AMPK		

hMSCs, human mesenchymal stem cells; WAT, white adipose tissue; BAT, brown adipose tissue; UCP1, uncoupling protein-1; PGC-1α, peroxisome proliferator-activated receptor-gamma coactivator-1-alpha; CIDEA, cell-death-inducing DFFA-like effector-a; Sirt3, sirtuin 3; NRF1, nuclear respiratory factor 1; p-AMPK, phosphorylated AMP-activated protein kinase; p-ACC, phosphorylated acetyl-CoA carboxylase; PPARγ, peroxisome proliferator-activator receptor gamma; C/EBPα, CCAT/enhancer-binding protein alpha; PRDM16, PR domain zinc finger protein 16; TBX1, t-box protein 1; PDK4, pyruvate dehydrogenase lipoamide kinase isozyme 4; COX8β, cytochrome *c* oxidase subunit 8B; EGCG, epigallocatechin gallate; cAMP, cyclic AMP; CPT-1, carnitine palmitoyltransferase; Dpt, dermatopontin; Igf, insulin-like growth factor; HSP60, heat shock protein 60; cyto c, cytochrome c; NADH, nicotinamide adenine dinucleotide; ND, not determined.

**Table 2:** Plant extracts to induce adipocyte browning.

Extract (Part/Solvent)	Model	Conc.	Effects	Active Component	Ref.
*Panax ginseng* (root and leaf/ND)	3T3-L1 cells	25–100 μg/mL	↓adipogenesis (↓C/EBPα, SREBP1-c) ↑mitochondrial activity ↑brown-adipocyte-specific markers (↑UCP1, PRDM16, PGC-1 α) ↑p-AMPK	Gensenoside Rb1 Rb2 Rg1 Rg3	[39,40,41,42,43]
	primary white adipocytes				
*Phyllostachys pubescens* and *Scutellaria baicalensis* (root and leaf/70% EtOH)	3T3-L1 cells	60–480 μg/mL	↓adipogenesis (↓PPARγ, C/EBPα) ↓lipogenesis (↓SREBP-1c, FAS) ↑fatty acid oxidation, lipolysis (↑p-ACC, CPT1) ↑BAT markers (↑UCP1, PRDM16, PGC1α) ↑thermogenesis (↑*UCP2*) ↑p-AMPK	Chlorogenic acid Orientin Isoorientin Baicalin Wogonoside Baicalein Tricin Wogonin Chrysin	[26]
*Humulus japonicas* (leaf/water)	3T3-L1 cells	20, 100 μg/mL	↑thermogenesis, browning (↑UCP1, PRDM16, PGC-1 α) ↑fatty acid oxidation, lipolysis ↓lipogenesis, lipid accumulation ↓oxidative stress/↑SOD1, catalase, GPx1 ↑AMPK/PPARδ signaling pathway	ND	[44]
Immature *Citrus reticulate* (fruit/hot water)	C57BL/6 mice	1%	↓body/visceral fat weights, adipocyte size ↓fatty livers, insulin resistance, dyslipidemia ↑cold tolerance in cold exposure ↑thermogenesis (↑UCP1, PRDM16, NRF1) ↑beige adipocyte-selective markers (↑TEME26, CD137, Cidea)	Synephrine Narirutin Hesperidin Nobiletin tangeretin	[45]
Broccoli Seeds (sprout/water)	C57BL/6JSlc mice	0.3%	↓body weight gain, fat mass ↑energy expenditure ↑major browning marker (↑UCP1) ↓insulin resistance, glucose tolerance ↓plasma lipopolysaccharide ↓relative abundance of Desulfovibrionaceae bacteria ↓oxidative stress, inflammation	Glucoraphanin	[46]

C/EBPα, CCAT/enhancer binding protein alpha; SREBP1-c, sterol regulatory element-binding protein 1; UCP1, uncoupling protein 1; PRDM16, PR domain zinc finger protein 16; PGC-1α, peroxisome proliferator-activated receptor gamma coactivator-1-alpha; p-AMPK, phosphorylated AMP-activated protein kinase; CIDEA, cell-death-inducing DFFA-like effector-A; HSP60, heat shock protein 60; cyto c, cytochrome c; NADH, nicotinamide adenine dinucleotide; WAT, white adipose tissue; Sirt1, sirtuin 1; SOD1, superoxide dismutase 1; GPx1, glutathione peroxidase 1; PPARδ, peroxisome proliferator-activated receptor delta; NRF1, nuclear respiratory factor 1; TEME26, transmembrane protein 26; CD137, cluster of differentiation 137; FAS, fatty acid synthase; p-ACC, phosphorylated acetyl-CoA carboxylase; CPT-1, carnitine palmitoyltransferase; BAT, brown adipose tissue; ND, not determined.
